# Removal and Reoccurrence of LLZTO Surface Contaminants under Glovebox Conditions

**DOI:** 10.1021/acsami.4c00444

**Published:** 2024-05-16

**Authors:** Marco Siniscalchi, Joshua S. Gibson, James Tufnail, Jack E. N. Swallow, Jarrod Lewis, Guillaume Matthews, Burcu Karagoz, Matthijs A. van Spronsen, Georg Held, Robert S. Weatherup, Chris R. M. Grovenor, Susannah C. Speller

**Affiliations:** † Department of Materials, University of Oxford, Oxford OX1 3PH, U.K.; ‡ The Faraday Institution, Didcot OX11 0RA, U.K.; § School of Chemistry, University of Edinburgh, Edinburgh EH9 3FJ, U.K.; ∥ Diamond Light Source, Didcot OX11 0DE, U.K.

**Keywords:** solid-state battery, LLZTO electrolyte, metal anode, interface, NAP-XPS

## Abstract

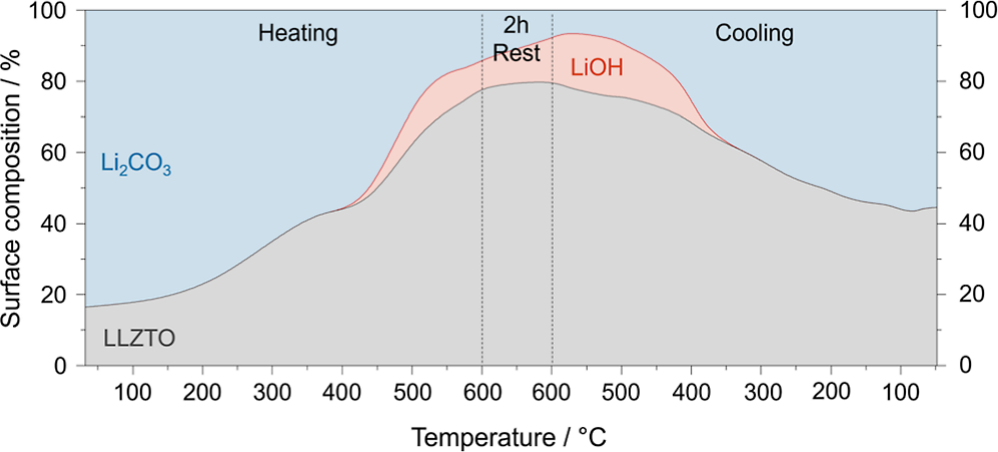

The reactivity of Li_6.4_La_3_Zr_1.4_Ta_0.6_O_12_ (LLZTO) solid electrolytes to form lithio-phobic species such as Li_2_CO_3_ on their surface when exposed to trace amounts of H_2_O and CO_2_ limits the progress of LLZTO-based solid-state batteries. Various treatments, such as annealing LLZTO within a glovebox or acid etching, aim at removing the surface contaminants, but a comprehensive understanding of the evolving LLZTO surface chemistry during and after these treatments is lacking. Here, glovebox-like H_2_O and CO_2_ conditions were recreated in a near ambient pressure X-ray photoelectron spectroscopy chamber to analyze the LLZTO surface under realistic conditions. We find that annealing LLZTO at 600 °C in this atmosphere effectively removes the surface contaminants, but a significant level of contamination reappears upon cooling down. In contrast, HCl_(aq)_ acid etching demonstrates superior Li_2_CO_3_ removal and stable surface chemistry post treatment. To avoid air exposure during the acid treatment, an anhydrous HCl solution in diethyl ether was used directly within the glovebox. This novel acid etching strategy delivers the lowest lithium/LLZTO interfacial resistance and the highest critical current density.

## Introduction

Solid-state batteries have emerged as a promising alternative to conventional lithium-ion batteries due to their higher energy density, enhanced safety, and potential for use in various applications, such as electric vehicles and portable electronics.^1^ The key component in solid-state batteries is the lithium-ion-conducting solid electrolyte (SE), which enables efficient lithium-ion transport between the electrodes. Among the various SEs, Li_6.4_La_3_Zr_1.4_Ta_0.6_O_12_ (LLZTO) stands out due to its relatively high room temperature ionic conductivity of ∼1 mS cm^–1^ and superior electrochemical stability when in contact with lithium metal.^2,3^

Initially, LLZTO was believed to be chemically stable when exposed to ambient air, a characteristic that could significantly benefit manufacturing processes and has contributed to the prominence of LLZTO.^4^ However, recent research has revealed that LLZTO, similar to several other lithium garnet oxides, undergoes a chemical reaction with H_2_O and CO_2_ in ambient air, leading to the formation of a surface contamination layer primarily comprising Li_2_CO_3_ and LiOH.^5,6^ This may occur through an initial reaction with moisture, where protons infiltrate the LLZTO lattice and substitute for Li^+^ in the tetrahedral sites to form LiOH. Subsequently, LiOH absorbs CO_2_ to form Li_2_CO_3_.
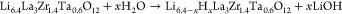
1
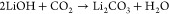
2

Another proposed reaction pathway involves direct reaction of LLZTO with CO_2_, although most studies tend to support a two-step mechanism, with water having a catalytic effect.^7−9^

Regarding the reaction rate, Leng et al. revealed that an untreated LLZTO surface, when exposed to air for a mere 24 h, develops a layer of contamination that is 120–160 nm thick.^10^ The reaction follows a diffusion-limited process; as the contamination layer thickens, the reaction rate decreases, and eventually, the reaction may come to a halt.^11^ Surprisingly, it has come to light that even in the supposedly inert atmosphere of an Ar-filled glovebox, an environment commonly used for the fabrication and storage of LLZTO cells in research settings, the formation of surface Li_2_CO_3_ and LiOH is still a concern. Despite the stringent efforts to maintain ultralow moisture levels (<0.1 ppm of H_2_O) in gloveboxes, background trace levels of moisture can be higher due to routine glovebox usage. Additionally, the concentration of CO_2_ in gloveboxes is generally not actively controlled or filtered. We assessed CO_2_ levels across various gloveboxes used for this work, utilizing a nondispersive infrared CO_2_ sensor (Analox), and found that CO_2_ concentrations ranged from 1 to 7 ppm. These seemingly negligible levels of H_2_O and CO_2_ are adequate to instigate the formation of a contamination layer on the LLZTO surface within a remarkably short span. For instance, Yamada et al. found that a clean LLZTO surface can accumulate a layer of contamination measuring 4–6 nm within as little as 30 min of exposure to a glovebox environment with <0.5 ppm of H_2_O and <5 ppm of CO_2_.^12^ Although the glovebox storage does mitigate the reaction kinetics to some extent, it is evident that the contamination process persists, albeit at a somewhat reduced rate compared to open-air exposure.

The presence of Li_2_CO_3_ and LiOH contaminants on the surface of LLZTO poses a substantial challenge to battery performance. First of all, these contaminants have lower ionic conductivity compared to LLZTO, thereby impeding ion transport across the electrode/LLZTO interface.^13^ Furthermore, in the context of solid-state cells, the LLZTO is commonly paired with a lithium metal anode. The contaminants exhibit lithiophobic characteristics, leading to incomplete contact between the LLZTO and the lithium metal electrode.^14^ Both the low ionic conductivity and lithiophobicity of the contamination layer result in a large lithium/LLZTO interfacial resistance *R*_int_. As a direct consequence, during cycling, the local current density at the active regions of the Li/LLZTO interface becomes much larger than the nominal applied value. This local current density concentration can result in adverse effects, such as cell polarization and formation of lithium dendrites through the SE,^15,16^ inevitably compromising the overall battery performance.

Hence, an efficient removal of surface contaminants is essential to achieve high-performance LLZTO-based solid-state batteries. Several surface treatment methods, such as mechanical polishing, annealing, and acid etching, have proven to be successful in removing the contamination layer, each exhibiting varying levels of effectiveness.^9^ A mechanical polishing step, either in air or in a glovebox, is often performed to obtain a uniform SE surface prior to cell assembly. While this step can reduce the surface contaminants, it cannot completely eliminate them.^6,14^ This could be due to the presence of contaminants along LLZTO grain boundaries, as observed through scanning electron microscopy analysis,^5^ which are not removed by a simple polishing step. Therefore, polishing is routinely followed by annealing or acid treatment.

Annealing within a controlled Ar atmosphere of a glovebox has been widely adopted in the literature. LiOH is removed at temperatures up to 500 °C by reversing eq 1, while Li_2_CO_3_ decomposes into Li_2_O and CO_2_ mostly around and above 700 °C.^10,14,17^ The precise annealing temperature required for the complete contamination removal depends on various LLZTO characteristics, for instance, doping elements and grain size, as well as on the annealing environment. Under ultrahigh vacuum (UHV) conditions, where materials volatilize at lower temperatures, annealing at 500–600 °C can almost completely remove the surface contaminants.^3,18,19^ However, a significant drawback of the heat treatment lies in the potential evaporation of lithium that can trigger the formation of pyrochlores such as La_2_Zr_2_O_7_ on the pellet surface, which possess a lower ionic conductivity.^20,21^ An alternative to annealing is acid etching, particularly appealing due to its simplicity and efficiency.^22−24^ A rapid immersion in a dilute aqueous acid solution such as HCl_(aq)_ can successfully decompose Li_2_CO_3_ according to
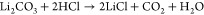
3

Following annealing or acid etching, however, there can be a resurgence of contamination before the Li/LLZTO interface is assembled, a point that has frequently been overlooked in the literature. This can occur even during short storage times in the glovebox before cell assembly^3,12^ and also during cooling from the annealing temperature back to room temperature or during the brief contact to air after the acid treatment in HCl_(aq)_ outside the glovebox. During these transitions, LLZTO can be exposed to H_2_O and CO_2_ leading to undesired surface reactions. Consequently, even after these treatments, residual interfacial resistance persists at the Li/LLZTO interface.

Adding complexity to the situation, LLZTO is often transferred from the glovebox to various instruments for further characterization using an O-ring-sealed transfer vessel without independent pumping. Unfortunately, this vessel may allow air leakage or gas desorption from the vessel walls even over a short period of time.^25,26^ As a result, any LLZTO surface analyzed after such a transfer is likely to exhibit additional surface LiOH and Li_2_CO_3_, formed during the transfer process. This intricacy clouds the accurate determination of the origin of these surface contaminants, whether they originated after the surface treatment within the glovebox or during the transfer process. This highlights the necessity for in situ characterization techniques that can provide a more precise understanding of the LLZTO surface reactivity.

This work focuses on the investigation and comparison of annealing and acid etching treatments. To accurately study the LLZTO surface chemistry under realistic conditions, we employ in situ near ambient pressure X-ray photoelectron spectroscopy (NAP-XPS) and simulate a glovebox-like environment by dosing H_2_O vapor and CO_2_ gas into the analysis chamber. This introduces a new opportunity to monitor in real-time the evolution of LLZTO surface chemistry during and after various surface treatments directly within the atmosphere where LLZTO is commonly treated and stored. We find that a heat treatment at 600 °C under glovebox-like H_2_O and CO_2_ contaminant levels is effective in removing the surface contaminants, but upon cooling back to room temperature, they promptly reappear as the clean LLZTO surface quickly reacts with H_2_O and CO_2_ at 300–400 °C. The final amount of contamination on the LLZTO is nonetheless lower than after a simple polishing step in air. The cooling behavior of LLZTO has frequently been neglected in previous investigations of surface treatments. However, understanding this aspect is crucial to the efficacy of the annealing treatment.

**Figure 1:**
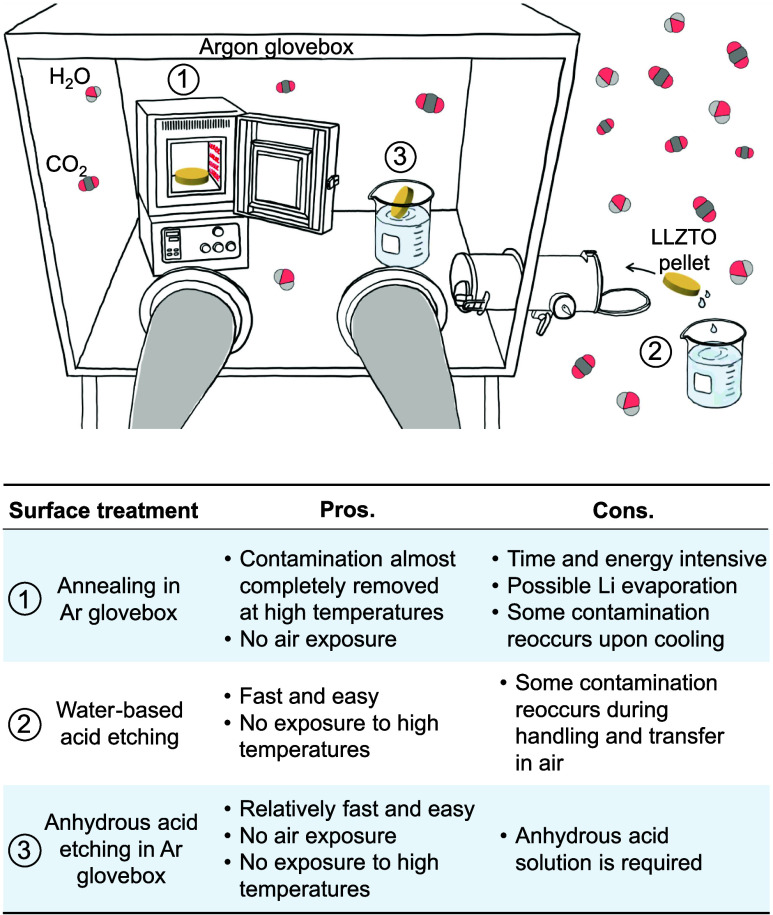
Overview of the treatments for the removal of surface contamination on LLZTO SEs investigated in our study. The table highlights the advantages and disadvantages of each treatment. Acid etching in the Ar glovebox with an anhydrous solution combines the advantages of the other two methods.

We also find that a rapid acid etch with HCl_(aq)_ can prove more effective in removing the surface contaminants than the annealing treatment if the LLZTO pellet is quickly brought into the glovebox environment after the HCl_(aq)_ etch, which is necessarily carried out in air. Moreover, the surface chemistry after etching remains stable in the glovebox-like atmosphere long enough to assemble the Li/LLZTO interface. To avoid the short exposure to air after the acid treatment, we have further developed a novel anhydrous acid treatment with a solution of HCl in diethyl ether () that can be conducted inside a glovebox. As illustrated in Figure 1, this new method combines the advantages from the other two methods, namely, no exposure to air nor to high temperatures. The interfacial resistance between lithium and LLZTO, which correlates with the amount of lithiophobic Li_2_CO_3_ and LiOH on the LLZTO surface, is minimized by the new  treatment.

## Results and Discussion

### Annealing in a Glovebox-Like Environment

Annealing LLZTO within the controlled atmosphere of a glovebox has been widely acknowledged as an effective strategy for the removal of surface Li_2_CO_3_ and LiOH.^10,14,17^ Nevertheless, this approach is prone to reformation of the contaminants during the cooling phase, as elucidated in this section.

**Figure 2:**
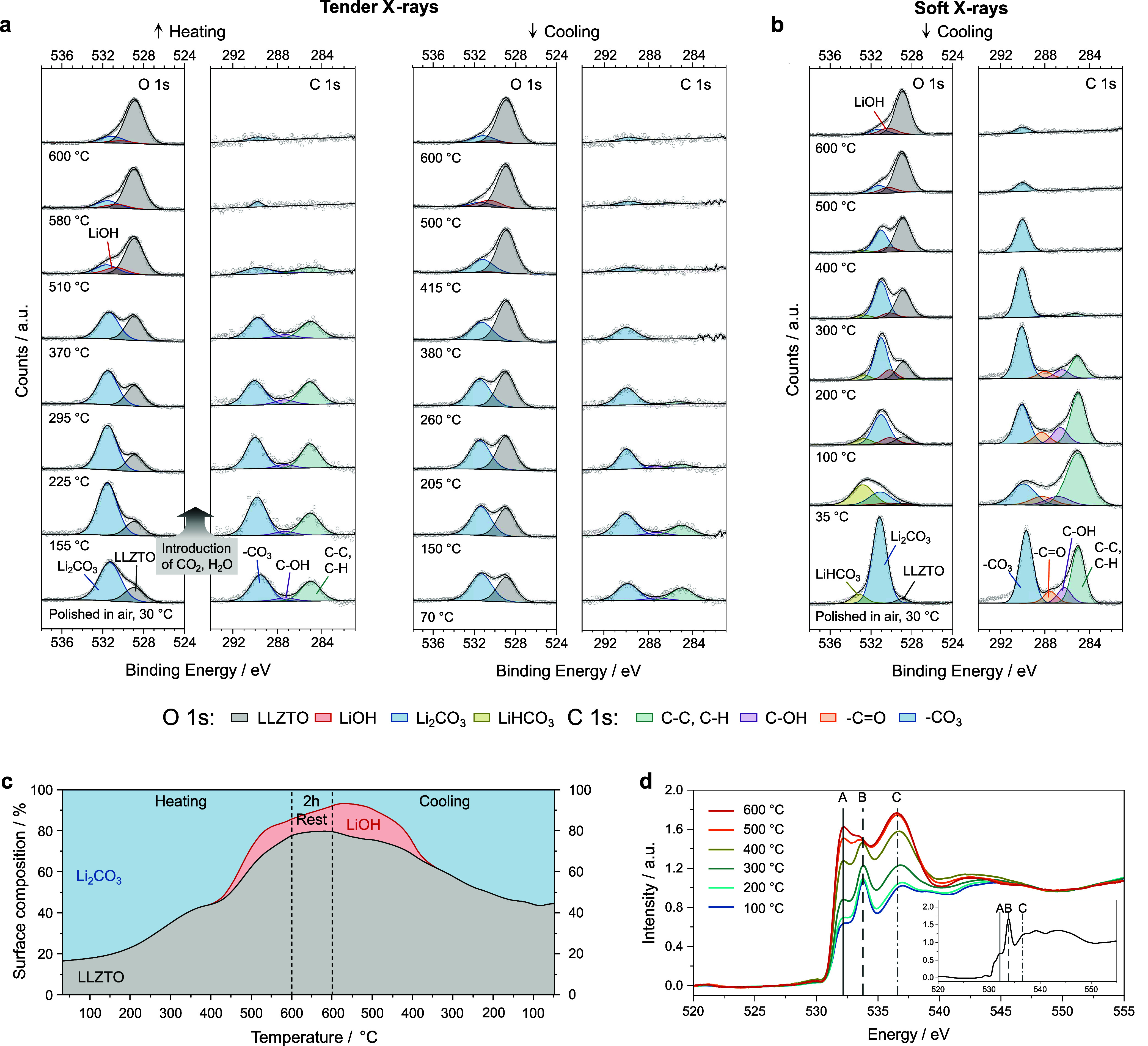
(a) O 1s and C 1s XPS spectra collected with tender X-rays showing the removal and reoccurrence of contamination during a full annealing treatment of LLZTO. The bottom spectra on the left correspond to the as-loaded polished pellet analyzed under UHV conditions and at room temperature. The subsequent spectra are collected while heating and cooling the same sample in a glovebox-like atmosphere (corresponding to 1 ppm of H_2_O, 4 ppm of CO_2_). (b) Soft XPS signals collected every 100 °C upon cooling the LLZTO pellet back to room temperature. The bottom spectra correspond to the as-loaded surface signals. (c) Evolution of the relative concentration of surface contaminants with temperature, obtained from the tender X-ray XPS signals coming from the top ∼ 15 nm of the surface. (d) O K-edge near edge X-ray absorption fine structure (NEXAFS) spectra for an LLZTO pellet during cooling from 600 °C in a glovebox-like atmosphere. Key spectral features are highlighted as A (LLZTO), B (Li_2_CO_3_), and C (LLZTO). Inset: spectrum for an as-loaded LLZTO pellet, with the same features highlighted.

The LLZTO pellet was polished in air and promptly introduced into the sample load-lock before transferring to the NAP-XPS chamber (base pressure of ∼3 × 10^–8^ mbar) such that the pellet goes from air to UHV in about 60 s. The O 1s and C 1s XPS signals were collected to study the evolution of surface chemistry throughout the annealing treatment. X-rays with an incident energy of 2500 eV (subsequently referred to as tender X-rays for simplicity) were employed to probe deeper surface layers with a probing depth from approximately 10 to 15 nm. During cooling, softer X-rays were also used, tuning the incident energy to achieve a kinetic energy of the ejected photoelectrons of ∼315 eV and probing the topmost ∼3 nm of the LLZTO surface. The initial O 1s and C 1s spectra under UHV conditions at 30 °C are reported at the bottom of Figure 2a and b for the tender and soft X-rays, respectively.

Considering the tender X-ray data, the survey spectrum reported in Figure S1, the O 1s spectrum at the bottom of Figure 2a exhibits a large peak (B.E. ∼ 531.4 eV) with a low-energy shoulder attributed to LLZTO lattice oxygen (B.E. ∼ 528.9 eV). This shape correlates well with the previously published data where, in many cases, the high-energy peak has been fitted with both Li_2_CO_3_ and LiOH with a peak separation of ∼0.9 eV.^3,8^ In our case at 30 °C, the LiOH peak has zero intensity, suggesting that after polishing in air, the primary surface impurity is Li_2_CO_3_. The C 1s spectrum is fitted with –CO_3_, C–OH, and adventitious C–C/C–H peaks at ∼289.6, 287.3, and 285 eV respectively. Fitting constraints used in this study, which are based on the existing literature, are reported in the Experimental Section. Following the preliminary characterization in UHV, a glovebox-like environment was reproduced in the NAP-XPS chamber by introducing small quantities of CO_2_ gas (4 × 10^–3^ mbar, corresponding to 4 ppm when diluted in Ar at 1 bar) and H_2_O vapor (1 × 10^–3^ mbar, corresponding to 1 ppm in Ar at 1 bar). The LLZTO pellet was then heated to 600 °C at a rate of 10 °C/min. Figure 2a shows the most significant changes to the O 1s and C 1s spectra during heating (bottom to top) and cooling (top to bottom). The evolution of LLZTO, Li_2_CO_3_, and LiOH signals can also be visualized in Figure 2c, which displays the percentage of surface oxygen species calculated from the peak areas. During the heating step, the O 1s spectrum shows an increase in the LLZTO lattice oxygen signal, while the surface Li_2_CO_3_ is gradually removed with temperature. The signal evolves to some extent at also lower temperatures, although the primary temperature window for Li_2_CO_3_ removal is in the range of 400–500 °C. This is validated by the C 1s signal, where the –CO_3_ peak essentially disappears around those temperatures. The intensity of the C–C/C–H peak also decreases alongside the –CO_3_ signal, meaning that adventitious carbon species are also leaving the LLZTO surface.

Above 400 °C, a small LiOH peak at ∼530.5 eV becomes evident within the O 1s spectrum. It is important to emphasize that an inherent dynamic equilibrium exists between the decomposition and reformation processes of surface contaminants. The direction and kinetics of reactions 1 and 2 are dependent not only on the temperature but also on the availability of reactants. As Li_2_CO_3_ starts to display considerable decomposition above 400 °C, the pristine LLZTO surface comes into contact with the H_2_O and CO_2_ introduced into the NAP-XPS chamber. Under these specific conditions, LLZTO appears to react with H_2_O resulting in the formation of a minor quantity of LiOH. However, this LiOH does not seem to react with CO_2_ to increase the amount of surface Li_2_CO_3_, which instead keeps decreasing. At 600 °C, the O 1s spectrum shows a prevalent LLZTO peak with negligible Li_2_CO_3_ and LiOH signals, whereas the C 1s signal assumes a mostly flat profile. Therefore, for this study, we consider annealing at 600 °C to be sufficient to achieve a clean LLZTO surface. The minor remaining contamination may be mainly associated with grain boundaries.^5^ However, we avoided annealing at higher temperatures to prevent excessive vaporization of the lithium metal from the near-surface regions, as suggested in previous reports.^21^

The sample was left at 600 °C for 2 h, after which it was gradually cooled back to the room temperature. As the temperature decreases from 500 to 350 °C, the intensity of the LiOH signal diminishes, while the Li_2_CO_3_ signal grows back. This could be attributed to the reaction between LiOH and CO_2_ in eq 1. The Li_2_CO_3_ signal gradually intensifies as the temperature decreases to 200 °C, below which all of the signals are quite stable. The –CO_3_ peak as well as the adventitious C–C/C–H peak in the C 1s signal also re-emerge. The latter resurgence may be attributed to the redeposition of carbonaceous contaminants from the NAP-XPS chamber, a phenomenon that we believe would be even more pronounced in a real glovebox environment.

While the sample was left to cool, soft X-ray spectra were also collected at every 100 °C intervals to elucidate the evolution of the topmost 3 nm of the LLZTO pellet (Figure 2b). The data obtained from soft X-rays largely corroborate the findings obtained from tender X-rays. At 600 °C, the LLZTO surface features only minor LiOH and Li_2_CO_3_ peaks. Upon cooling, the LiOH signal remains relatively small, while the Li_2_CO_3_ peak grows stronger in intensity. At around 200 °C, attenuation of the LLZTO and LiOH peaks is interpreted as Li_2_CO_3_ covering the majority of the topmost surface. Below 200 °C, an additional peak emerges in the O 1s spectrum at a higher binding energy of ∼532.8 eV. We assign this peak to the hydrated form of Li_2_CO_3_, lithium bicarbonate (LiHCO_3_), which can form according to
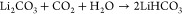
4

Xu et al. showed that this reaction occurs below 85 °C.^27^ It is possible that during cooling, the topmost LLZTO surface is at a slightly colder temperature than that of the bulk, justifying the appearance of the LiHCO_3_ peak at higher sample bulk temperatures. The monohydrated form of LiOH might also exist as LiOH·H_2_O,^10,28−30^ but an additional peak was not used in the fitting model. The LiHCO_3_ peak was also visible in the spectrum after polishing LLZTO in air, reported at the bottom of Figure 2b, which shows a predominant Li_2_CO_3_ peak. It is reasonable to presume that both LiHCO_3_ and Li_2_CO_3_ gradually disappear from the topmost LLZTO surface during heating at a rate similar to that observed with tender X-rays. After cooling to temperatures below 100 °C, LiHCO_3_ and Li_2_CO_3_ completely cover the LLZTO surface. Pristine LLZTO is still present beneath the topmost surface as shown by the tender X-ray data. The C 1s soft X-ray spectrum similarly grows back during cooling and can be fitted by a four-peak model, including a –C=O peak that we attribute to adventitious contamination. We do not observe changes in the C 1s spectra that can be attributed to a LiHCO_3_ peak, which is likely masked under the Li_2_CO_3_ peak.

O K-edge NEXAFS data were also collected during cooling from 600 °C and are reported in Figure 2d. These were collected at 100 °C decrements in-step with the XPS measurements shown in the other panels of Figure 2. We utilized the surface-sensitive total electron yield mode, giving a probing depth of <10 nm.^6^ Three major features are visible in the K-edge spectra at 532.2 eV (A, solid line), 533.8 eV (B, dashed line), and 535.8 eV (C, dotted-dashed line).

The spectrum of the as-loaded LLZTO (Figure 2d—inset) is dominated by a sharp peak at B, corresponding to transitions from the O 1s to carbonyl (C=O) π* orbital of Li_2_CO_3_. Peaks A and C primarily correspond to transitions to O 2p–Me 4d/5d hybridized states of LLZTO with e_*g*_ and t_2*g*_ symmetry, respectively.^6,31−34^ At 600 °C, peaks A and C are dominant, consistent with much of the Li_2_CO_3_ having been removed. The small feature close to peak B occurs at a slightly lower energy than seen for Li_2_CO_3_, consistent with the presence of LiOH.^35^ On cooling, the growth in the B peak, its shift to slightly higher energy, and the lowering in the intensity of peaks A and C are consistent with the increasing coverage of the LLZTO with Li_2_CO_3_, particularly below 400 °C. This further reinforces the interpretation of the LLZTO surface evolution obtained from the XPS measurements.

**Figure 3:**
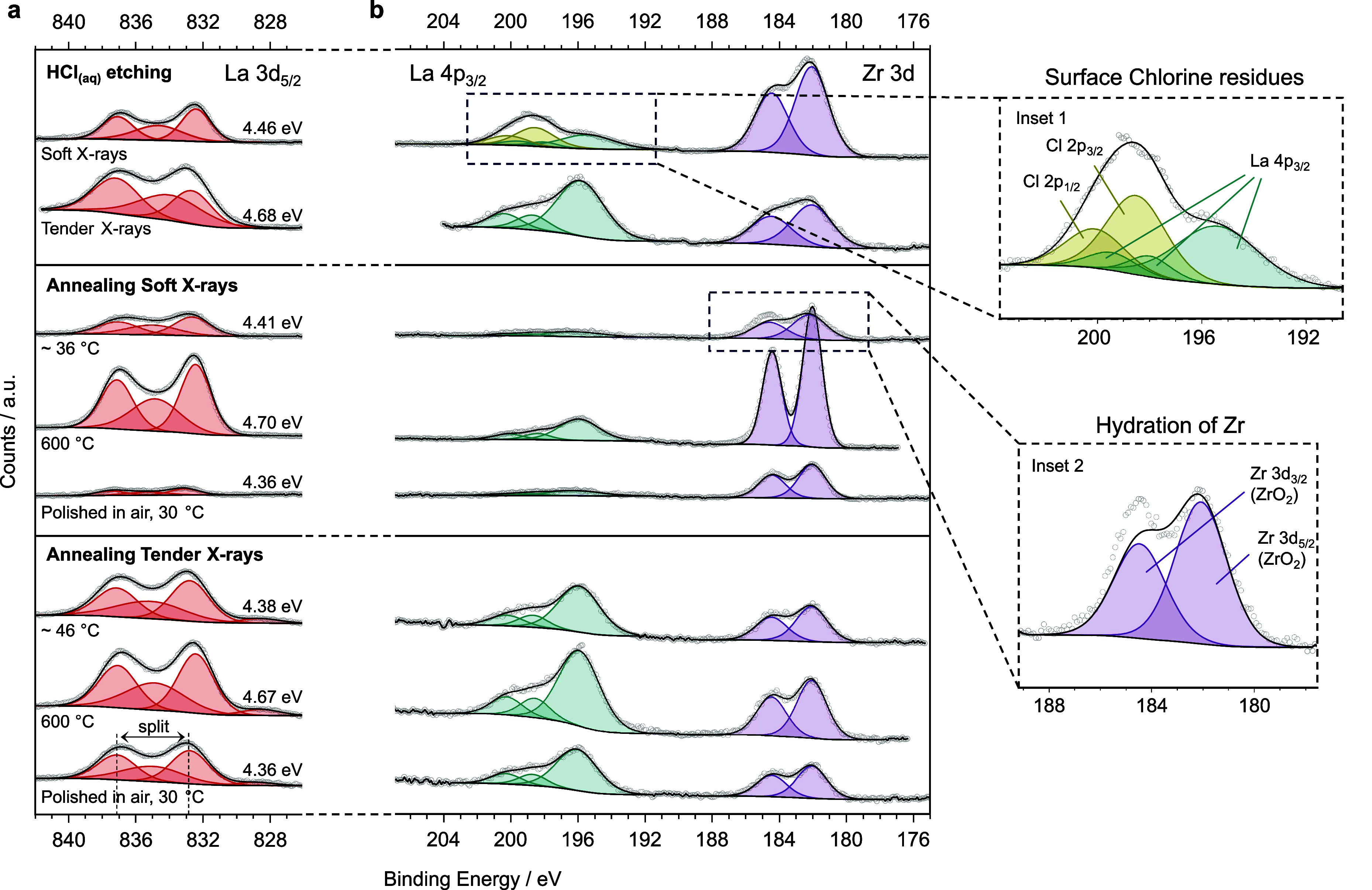
(a) La 3d_5/2_ XPS spectra collected from LLZTO pellets at different stages of the surface treatments. From bottom to top, annealing tender X-ray spectra, annealing soft X-ray spectra, and HCl_(aq)_ etching spectra. The values of the La 3d_5/2_ multiplet energy splitting, which give indication of the oxidation state of La, are reported above each spectrum on the right. (b) La 4p_3/2_ and Zr 3d XPS spectra. The soft X-ray spectrum collected after HCl_(aq)_ etching contains a contribution from Cl 2p, shown in detail in inset 1. Inset 2 reports the change in the soft X-ray Zr 3d signal after annealing. The change can be explained by a slight hydration of Zr.

For a more comprehensive characterization, La 3d_5/2_, La 4p_3/2_, and Zr 3d XPS spectra were collected directly after LLZTO polishing, at 600 °C and once more after cooling to room temperature. Both the tender and soft X-ray data are shown in the two bottom panels of Figure 3. The La 3d_5/2_ multiplet splitting can offer some insights into the oxidation state of La.^18,36^ The existing literature suggests a multiplet split between 3.7 and 4.1 eV for La(OH)_3_ and La carbonates and between 4.3 and 4.9 eV for La_2_O_3_.^37,38^ As reported in Figure 3a, the split before annealing is 4.36 for both tender and soft X-rays, suggesting the presence of a mixture of La oxide and hydroxide/carbonate environments. At 600 °C, when the LLZTO surface is mainly free of contaminants, the splitting increases to approximately 4.7 eV. This value more closely aligns with that of La_2_O_3_, indicative of La in the chemical environment of the LLZTO lattice. After cooling to room temperature, the splitting decreases to ∼4.4 eV. Therefore, the La 3d_5/2_ multiplet splitting qualitatively agrees with the evolution of surface contamination as suggested by the O 1s and C 1s signals.

As for the La 4p_3/2_ and Zr 3d spectra, Figure 3b shows that the tender X-ray spectra remain consistent during annealing. Interestingly, the soft X-ray Zr 3d spectrum slightly changes after the LLZTO pellet is cooled to room temperature. The high-energy peak, sitting at ∼184.4 eV, has increased in intensity. However, the ratio between Zr 3d_5/2_ and 3d_3/2_ is fixed at 3:2 due to the degeneracies of the spin–orbit split levels (see inset 2 in Figure 3b). Therefore, an additional high-energy contribution might be included in our data, which could be assigned to a hydrated form of ZrO_2_, as previously reported,^39^ although no satisfactory fitting could be achieved based on the constrained Zr 3d model. Hence, it is likely that upon cooling, the topmost surface layer of LLZTO evolves with a complex chemistry, including modifications to the La and Zr chemical environments. This could happen during the protonation of LLZTO, i.e., the H^+^/Li^+^ exchange as in eq 1. The O 1s spectra in Figure 2b may contain some minor contributions from La and Zr carbonates or hydroxyls, but these appear to be negligible or overlap with other O components. The relative peak intensity changes observed between tender and soft X-ray measurements for La 4p and Zr 3d are in line with the expected changes based on the relative X-ray photoionization cross sections.

In this section, we have demonstrated the efficacy of annealing at 600 °C in a glovebox-like environment in eliminating the surface Li_2_CO_3_ from a contaminated LLZTO surface. However, we have also shown that the cleaned LLZTO surface is susceptible to reaction with even low levels of H_2_O and CO_2_ at elevated temperatures, facilitating the re-emergence of surface contaminants upon cooling the LLZTO pellet to room temperature. Nonetheless, the quantity of surface Li_2_CO_3_ after cooling remains lower than the initial amount following polishing in air, explaining how annealing LLZTO can lead to reduced Li/LLZTO interfacial resistance. Next, we discuss the potential of acid etching to yield an even cleaner LLZTO surface. Notably, acid etching operates at room temperature, where a pristine LLZTO surface is less reactive with glovebox contaminants compared to its behavior at high temperatures during the annealing treatment.

### Acid Etching in HCl_(aq)_

Pioneering work by Huo et al. and subsequent studies have demonstrated the efficacy of rapid acid treatment in HCl_(aq)_ or H_2_SO_4(aq)_ to remove the surface Li_2_CO_3_, leading to an electrochemically more favorable Li/LLZTO interface.^22−24^ This treatment involves briefly immersing the LLZTO pellet in an aqueous acid solution, often followed by a washing step with alcohols to eliminate residual HCl from the surface. Subsequently, a quick drying step with blown air or inert gas is conducted before the introduction of LLZTO into the glovebox. The simplicity and efficiency of the rapid acid treatment make it particularly appealing. However, as this treatment entails contact between LLZTO and an aqueous solution followed by a short air exposure, there is a possibility of some surface contamination reforming on the pellet.^22,23^ Still, the treatment can be effective if the time taken to transfer LLZTO into the glovebox following the acid treatment is minimized. Furthermore, once the LLZTO is in the glovebox environment, its reactivity with H_2_O and CO_2_ at room temperature is reduced, as we will show in this section.

**Figure 4:**
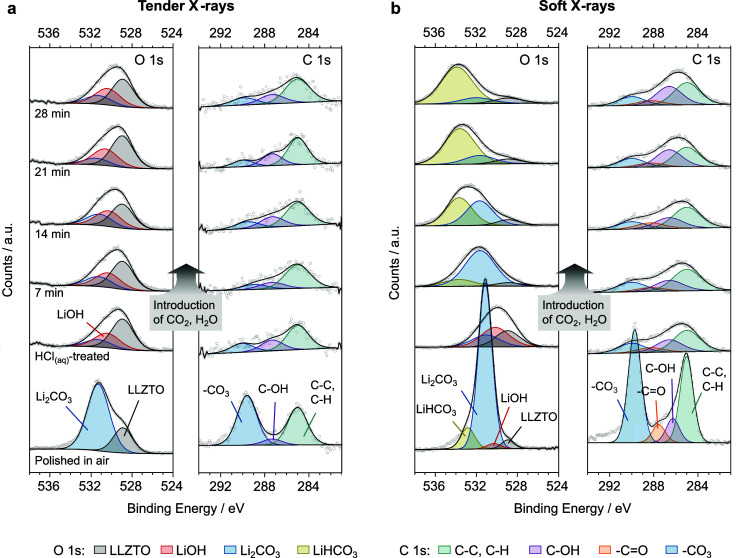
(a) Tender and (b) soft XPS O 1s and C 1s signals collected from LLZTO pellets following acid treatment in HCl_(aq)_. For comparison, the bottom spectra correspond to pellets polished in air. The spectra above them are collected first under UHV conditions and then in a glovebox-like atmosphere (corresponding to 1 ppm of H_2_O, 4 ppm of CO_2_).

To compare the efficiency of acid etching with that of the annealing treatment, we polished an LLZTO pellet in air and immersed it in 1 M HCl_(aq)_ for 10 s. The pellet was washed with ethanol and isopropanol and then dried with an Ar jet before loading it into the NAP-XPS system. Comparison of the O 1s and C 1s signals from the as-loaded LLZTO after acid etching (Figure 4) and after polishing in air (previously shown in Figure 2a and b, and now reported at the bottom of Figure 4) reveals a stark contrast. The tender X-ray O 1s data for the acid cleaned surface now exhibit a strong LLZTO peak with comparatively smaller Li_2_CO_3_ and LiOH peaks. This is corroborated by the C 1s signals, where the –CO_3_ peak is relatively small. Surface contamination, mainly LiOH, is more prominent in the soft X-ray data, but the LLZTO peak is still visible. The presence of LiOH, which is absent after polishing in air, is likely to originate from the contact of LLZTO with H_2_O in the aqueous acid solution.^40,41^ This likely leads to protonation of the LLZTO surface as well as the formation of LiOH, according to eq 1.

Moreover, the La 3d_5/2_ spectra in the top panel in Figure 3a show a multiplet splitting of 4.68 eV for the tender X-rays, suggesting that the acid etching can reveal a more pristine LLZTO surface. The splitting of 4.46 eV for the soft X-rays confirms that some contamination is present on the very topmost surface. The La 4p_3/2_ soft X-ray signal includes a doublet peak that can be assigned to Cl 2p (see inset 1 in Figure 3b). This peak is ascribed to the residual chloride anions which have not been rinsed away following etching and is only observed in minor quantities in the surface-sensitive soft X-ray data. XPS signals for the acid-treated LLZTO are slightly but consistently broader than the data collected on the annealed LLZTO. We believe this might be due to a reduced electronic charge compensation for the acid-treated sample.

After this preliminary characterization, 4 × 10^–3^ mbar of CO_2_ and 1 × 10^–3^ mbar of H_2_O were dosed into the NAP-XPS chamber to simulate the introduction of the acid-treated LLZTO pellet into a glovebox. XPS measurements were conducted at 7 min intervals for nearly 30 min, which would be enough time to assemble the Li/LLZTO interface in the glovebox. Minimal changes are observed in the more bulk-sensitive O 1s and C 1s tender X-ray spectra during this period. Therefore, these results suggest that the LLZTO underneath the topmost surface remains relatively free from contamination, following introduction into a glovebox environment. However, some change is visible in the top 3 nm of the LLZTO surface during storage in the glovebox-like environment. As soon as CO_2_ and H_2_O are introduced in the NAP-XPS chamber, the LiOH peak, visible in the low photon energy O 1s spectrum for the as-loaded LLZTO, disappears in favor of a large Li_2_CO_3_ peak. This indicates that the topmost surface of LLZTO quickly contaminates within a glovebox environment as LiOH reacts to form Li_2_CO_3_, according to eq 2. During continued gas exposure, the O 1s spectrum clearly changes shape again, and the Li_2_CO_3_ decreases in intensity, while a peak at even higher binding energy progressively overshadows the other signals. As previously described, we assign this peak to LiHCO_3_. The resulting surface spectrum after 30 min of glovebox storage resembles that after cooling from the annealing treatment (Figure 2b). Although we would expect the –CO_3_ peak to increase in the soft X-ray C 1s spectra, we do not observe significant changes during exposure to the glovebox-like atmosphere. It is possible that the –CO_3_ and –HCO_3_ signals are not distinguishable here and that the amount of surface carbonates remains more or less constant with time, but further data are required to shed light on this matter. Under these conditions, the LiHCO_3_ signal is not seen in the bulk data, suggesting bicarbonate growth only on the topmost surface of the LLZTO.

The XPS data presented in this section suggest that acid etching LLZTO in HCl_(aq)_ cannot achieve the same degree of contamination removal that is achieved at 600 °C during the annealing treatment. However, considering that surface contamination reappears during cooling from 600 °C, acid etching actually proves to be more effective than the annealing treatment. Moreover, once acid-treated LLZTO is reintroduced into the glovebox environment, the growth of the surface contaminants occurs at a slow rate. In fact, the XPS signals from deeper LLZTO surface layers (∼15 nm) remain unchanged during 30 min of glovebox storage. Figure S2 compares the tender X-ray O 1s spectra from LLZTO pellets “just before cell assembly” within the glovebox, showing that the degree of surface contamination is lower for an acid-treated LLZTO pellet. The small amount of surface reaction on the acid-treated pellet might be due to the brief air exposure necessary to return the LLZTO to the glovebox-like atmosphere after immersion in the HCl_(aq)_ solution. In the next section, we demonstrate that the Li/LLZTO interface can be further improved by performing the acid treatment directly within the glovebox environment using an anhydrous acid solution.

### Acid Etching in 

To prevent air exposure of LLZTO following the acid treatment, we employed a solution of HCl in diethyl ether (), which is compatible for use within a controlled glovebox environment. Specifically, an LLZTO pellet was polished in air and promptly brought into a glovebox, where it was immersed in 1 M  for 10 min. Li_2_CO_3_ is more soluble in water than in ethers, possibly explaining the longer immersion time needed with .^42^ Subsequently, washing with dry ethanol and isopropanol was performed, followed by careful drying with an Ar jet.

In this section, we do not use NAP-XPS to track the evolution of the LLZTO surface chemistry subsequent to  etching. The reason for this lies in the fact that the NAP-XPS chamber was not directly connected to a glovebox where the  etching could be performed. Although sealed transfer vessels are frequently used to transfer samples between the glovebox and the XPS setup, we have observed that a significant additional surface contamination can develop during the transfer to the XPS in the sealed vessel, which complicates the surface analysis (Figure S3). The sensitivity of lithium SE surfaces to trace amounts of H_2_O and CO_2_ leaking in the transfer vessel has also been documented in a previous study by Gibson et al.^26^ Thus, the utilization of XPS was deemed inappropriate for investigating the  acid treatment. Instead, we have collected electrochemical impedance spectroscopy (EIS) of Li/LLZTO/Li cells to demonstrate the efficacy of  etching in eliminating surface contamination and preventing its reformation. EIS spectra were fitted to quantify the Li/LLZTO interfacial impedance, which is directly related to the degree of contamination present on the LLZTO surface.

**Figure 5:**
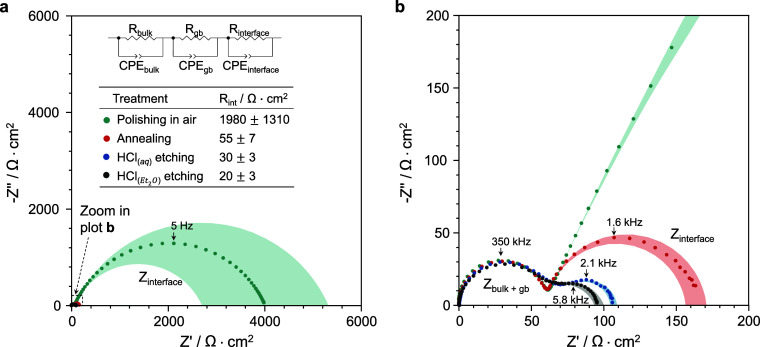
Electrochemical impedance spectra for the Li/LLZTO/Li cells assembled after LLZTO was subjected to the various surface treatments. The full low-frequency region is visible in plot (a), while plot (b) is a cut-out of the high-frequency region. The equivalent circuit model used to fit the spectra is shown in the inset of (a). The values of *R*_int_ (for a single Li/LLZTO interface) are also reported in the table. The shaded areas represent the error bounds calculated from at least three cells for each treatment.

The outcomes of our investigation are summarized in Figure 5, which shows the EIS spectra for the Li/LLZTO/Li cells. These cells were immediately assembled after the specific surface treatment to remove the LLZTO surface contaminants. To interpret the EIS data, we used a model comprising three RC elements, as shown in the inset of Figure 5a. Two RC elements are used to fit the high-frequency data, which probe the impedance inherent to the bulk LLZTO material and the LLZTO grain boundaries. The third element is suited for the low-frequency data, which represent the Li/LLZTO interface contribution. From these data, we quantified the resistance to lithium ion transport across a single Li/LLZTO interface and reported the values in the table in Figure 5a. At least three cells were assembled and tested for each surface treatment. A Li/LLZTO interface assembled with an LLZTO pellet subjected solely to polishing in air exhibits a *R*_int_ value of 1980 ± 1310 Ω cm^2^. The relatively large cell-to-cell variation suggests that it is difficult to obtain consistent Li/LLZTO interfaces without additional LLZTO surface treatment. The annealing treatment managed to significantly lower the *R*_int_ value to 55 ± 7 Ω cm^2^, while acid etching in HCl_(aq)_ yielded a value of 30 ± 3 Ω cm^2^. Notably, the acid treatment involving  resulted in the most favorable outcome, with the lowest interfacial resistance of 20 ± 3 Ω cm^2^. This discernible reduction in interfacial resistance is attributed to a lower degree of contamination present on the LLZTO surface. The *R*_int_ can be further reduced after cell assembly by heating the Li/LLZTO/Li cell to 170 °C for a few hours.^43^ This thermal conditioning softens the lithium metal electrodes, allowing them to flow into microscopic interfacial gaps, ultimately resulting in a near-zero interfacial resistance (Figure S4). This also suggests that the removal of contamination from the LLZTO surface was essentially complete.

**Figure 6:**
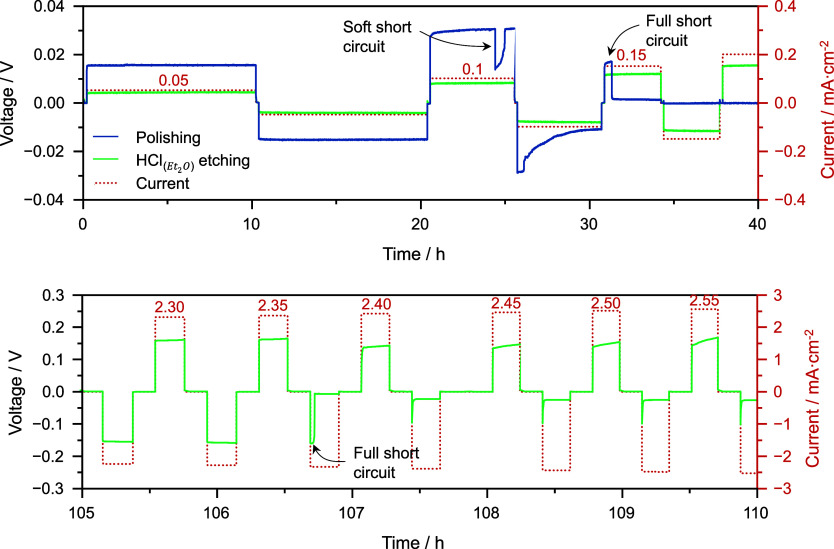
Critical current measurements of Li/LLZTO/Li cells. LLZTO underwent simple polishing (blue line) or  etching (green line). Both cells underwent a thermal preconditioning at 170 °C. The current density was increased by 0.05 mA cm^–2^ after each cycle, and a capacity of 0.5 mA h cm^–2^ was plated or stripped during each half cycle. The cells were cycled at 30 °C and with 3 MPa of external stack pressure. Lithium dendrites start forming in the as-polished LLZTO cell at 0.1 mA cm^–2^, and full short-circuiting is visible at 0.15 mA cm^–2^. The surface-treated LLZTO cell undergoes short-circuiting at 2.35 mA cm^–2^.

As is well established, a lower *R*_int_ invariably translates to improved cell performance,^15^ emphasizing the critical role that the Li/LLZTO interface plays in enhancing the overall efficiency of the battery. To demonstrate this point, critical current density measurements were conducted on Li/LLZTO/Li cells assembled with two different LLZTO pellets (Figure 6). The first LLZTO pellet received no surface treatment, while the other underwent  acid etching. Both Li/LLZTO/Li cells underwent subsequent preconditioning at 170 °C to improve the interfacial impedance. This test involves increasing the current density in a stepwise manner after each plating/stripping cycle until a short circuit arises due to lithium dendrite growth within the pellet. Notably, for the same applied current, the polarization of the cell differs due to the varying Li/LLZTO interfacial resistance. For the cell with as-polished LLZTO, lithium dendrites start growing at 0.1 mA cm^–2^ (soft short circuit), and the critical current density value, representing the current density at which lithium dendrite penetration causes complete cell failure, is 0.15 mA cm^–2^. This value increases to 2.35 mA cm^–2^, an order of magnitude higher, for the cell with acid-treated LLZTO. This cell recovers some of its polarization in the half cycle following the short circuit, which can be attributed to the partial shrinkage of the dendrite upon reversing the current direction.^44^ Nevertheless, the cell is essentially damaged. This straightforward critical current density experiment underscores the significance of careful Li/LLZTO interface assembly for the performance of solid-state cells.

## Conclusions

Our study assesses the effectiveness of different treatments in mitigating surface contamination on LLZTO solid electrolytes. Interestingly, annealing at high temperatures, the most widely employed method for removing the surface contaminants, shows a significant problem; the contamination promptly reforms as the LLZTO pellet cools back to room temperature.

In contrast, a rapid acid treatment in HCl_(aq)_ yields a cleaner LLZTO surface by maintaining the pellet at room temperature throughout the procedure, where the kinetics of the reaction between LLZTO and trace CO_2_ and H_2_O are slower. However, the brief exposure to air decreases the efficacy of the treatment, allowing some superficial contamination to reappear. Furthermore, it makes this treatment susceptible to differences in the air moisture and CO_2_ content, i.e., the LLZTO is exposed to an environment which is difficult to control. Therefore, we believe that carrying out the acid treatment directly in a glovebox proves to be a more effective strategy to remove the surface contaminants. The novel acid treatment using  within a glovebox shows superior results, demonstrating the lowest Li/LLZTO interfacial resistance among the tested methods. This indicates its effectiveness in removing surface Li_2_CO_3_ and LiOH and largely preventing their reformation. Thus, *R*_int_ values decrease as follows: annealing > HCl_(aq)_ > . Using  can be particularly relevant for industrial dry-room operations, where it could be used in place of a more time-consuming and costly high-temperature annealing step.

This study also highlights the significance of conducting in situ characterization of LLZTO surfaces and showcases the potential of NAP-XPS to provide insights into the time scales required for surface passivation within a glovebox environment. This information is important for optimizing the LLZTO treatment and storage protocols. The use of this analysis technique allowed us to capture in detail the evolution of the LLZTO surface chemistry. The XPS data reveal for instance a correlation between the growth of the Li_2_CO_3_ signal and the reduction of the LiOH signal. This trend suggests that the formation of Li_2_CO_3_ might follow the reaction path outlined in eq 2. Furthermore, we were able to capture the existence of an additional high binding energy peak forming on the topmost surface of LLZTO upon exposure to the glovebox environment. This peak has been assigned to lithium hydrocarbonate, potentially arising from the reaction between Li_2_CO_3_ and H_2_O. Over extended storage periods, this hydrocarbonate could grow in thickness and play an important role in the surface chemistry of LLZTO.

In summary, our findings point out the vulnerability of LLZTO SE surfaces to contamination, even in controlled environments conventionally considered inert, such as gloveboxes. Moreover, the recurring formation of surface contaminants after surface treatments should not be overlooked, in particular, during the cooling step after annealing. This could also raise issues during the sintering process of LLZTO powder to produce SE pellets as contamination accumulates during cooling. Optimizing protocols for treating and storing LLZTO is essential to minimize the impact of surface reactions, which have a substantial influence over the performance and reliability of LLZTO-based solid-state batteries.

## Experimental Section

### Sintering LLZTO Pellets

Li_6.4_La_3_Zr_1.4_Ta_0.6_O_12_ powder (99.9%, Ampcera) was cold-pressed in a graphite die and then spark-plasma sintered (Dr Fritsch DSP 507) into dense pellets at 1200 °C and 50 MPa for 5 min. X-ray diffraction (XRD) measurements were carried out using an Empyrean diffractometer with Cu K_α1_ radiation (Malvern Panalytical) to ensure phase purity of cubic LLZTO (Figure S5). Circular pellets were cut into smaller pieces using a low-speed diamond saw to ensure high sample consistency. Pellets ∼0.7 mm thick were used throughout the study. The pellets were polished in air with a diamond lapping film (1 μm) until the surface was visually shiny immediately prior to the surface treatment.

### Annealing Treatment

After polishing in air, the LLZTO pellet was promptly introduced into the NAP-XPS chamber, which was evacuated within 60 s. After an initial characterization under UHV (∼3 × 10^–8^ mbar), CO_2_ gas and H_2_O vapor were introduced in the NAP-XPS chamber. CO_2_ (99.9995% purity) was dosed through a mass flow controller at 0.22 sccm, corresponding to a constant chamber pressure of 4 × 10^–3^ mbar. H_2_O vapor was then immediately introduced through a manually controlled leak valve connected to a quartz tube containing water, until a total chamber pressure of 5 × 10^–3^ mbar was obtained. These pressures correspond to 4 ppm of CO_2_ and 1 ppm of H_2_O when diluted in 1 bar of Ar, which simulate typical values normally found in an operating glovebox, where LLZTO annealing treatments are usually carried out. Subsequently, the sample was heated to 600 °C at 10 °C/min, left for 2 h at 600 °C, and cooled down to room temperature. During cooling, soft XPS and NEXAFS data were also collected at every 100 °C. The temperature was monitored by using a thermocouple in contact with the sample holder surface. The data collection took ∼3 min for O 1s and 4 min for C 1s; therefore, the temperature reported for each spectrum corresponds to an average. To assemble the Li/LLZTO/Li cell, the polished LLZTO pellets were introduced into a glovebox (O_2_ and H_2_O ∼ 1 ppm, CO_2_ ∼ 4 ppm) and promptly heated to 600 °C at 10 °C/min in a box furnace (MTI), using an Al_2_O_3_ crucible cladded with gold foil. The pellets were left at 600 °C for 2 h, before cooling back to room temperature over a period of 4 h. The temperature profile was similar to that in the NAP-XPS.

### HCl_(aq)_ Etching

After polishing in air, the LLZTO pellet was immersed for 10 s in 1 M HCl_(aq)_. Washing steps were then performed with ethanol and isopropanol to remove any HCl residues, and the pellet was dried with an Ar jet. Then, it was immediately (<30 s) loaded in the XPS chamber. First, a full characterization under UHV conditions was performed. Then, CO_2_ and H_2_O were introduced into the XPS chamber following the above procedure. C 1s and O 1s spectra were collected every 7 min for at least 30 min. For the Li/LLZTO/Li cell assembly, the LLZTO pellet was quickly brought into the glovebox following the acid treatment (<30 s), and the cell was immediately assembled.

###  Etching

The LLZTO pellet was airpolished in air and brought into the glovebox. It was immersed in 1 M  for 10 min, washed with dry ethanol and isopropanol, and dried with an Ar jet. The Li/LLZTO/Li cell was immediately assembled.

### XPS Data Collection and Analysis

X-ray photoelectron spectra were collected at the VerSoX beamline (B07-C) of Diamond Light Source.^45^ La 3d, O 1s, C 1s, and Zr 3d spectra were measured with two different photon energies in order to achieve surface and bulk sensitivity. For the latter, the incident X-ray energy was 2500 eV for all spectra, which leads to information depths between 10 nm for La 3d (kinetic energy ∼ 1650 eV) and 15 nm for Zr 3d (kinetic energy ∼ 2300 eV), assuming a pristine LLZTO surface. For high surface sensitivity, the photon energy was tuned such that the kinetic energy of the emitted photoelectrons was always 315 eV (∼3 nm information depth). This corresponds to the following incident energies: La 3d 1160 eV, O 1s 850 eV, C 1s 600 eV, and Zr 3d 500 eV. The electron analyzer pass energy for high-energy electrons was 60 eV, while for the low energies, it was 30 eV. Survey measurements were conducted with a step size of 0.5 eV, while core level spectra had a step size of 0.1 eV.

The data analysis was carried out with CasaXPS.^46^ GL(30) line shapes were used to fit the peaks, and model constraints from the literature were applied as follows. For the O 1s spectra, the Li_2_CO_3_ peak was fixed at LiOH + 0.9 eV, while LiHCO_3_ was fixed at LiOH + 2.6 eV.^3,8^ La 3d_5/2_ and La 4p_3/2_ spectra were fitted with a three-peak model, according to Sunding et al.^36^ The area ratio of the main peak and bonding or antibonding components was kept the same for all spectra. The bonding and antibonding peaks were fixed to the same area. The Cl 2p surface contamination was fitted with a doublet peak. The Cl 2p_1/2_ area was constrained to be half of Cl 2p_3/2_, the doublet peaks had the same full width half maxima, and a splitting of 1.6 eV. Zr 3d spectra were fitted with a doublet peak with a 2.4 eV splitting and full width half maxima constrained to be equal, with an area ratio of 3:2 (3d_5/2_/3d_3/2_).^39^ A background subtraction was performed for a better visualization of the XPS data as the background intensity can vary depending on temperature and charging effects. The C 1s spectra were energy calibrated with reference to the C–C/C–H peak at 285.0 eV, while the O 1s spectra to the lattice oxygen peak at 528.9 eV.^8^

### NEXAFS Data Collection

NEXAFS data were also collected at the VerSoX B07–C beamline. The oxygen K-edge data were collected in the total electron yield mode over the 520–555 eV range. The data were plotted normalized in the Athena XAS software, setting *E*_0_ to the first peak of the derivative, and calculating pre- and post-edge lines from −13.4 to −3.4 and 15.0 to 21.6 eV from *E*_0_, respectively.

### Cell Assembly and Testing

Two-electrode cells were assembled immediately after the different surface treatments. 200 μm thick Li electrodes were scraped to remove the surface contamination and gently pressed onto the SE after applying 2 mm ø polyimide tape masks to ensure a constant electrode area. For the thermal conditioning step, Li/LLZTO/Li cells were left at 170 °C on a hot plate in the glovebox for 2 h under approximately 2 MPa of pressure. All cells were sealed in Mylar pouch bags with Cu current collectors and removed from the glovebox for testing. They were left for 24 h at 30 °C within a temperature control chamber and with 3 MPa of external stack pressure. Then, the stack pressure was released, and potentiostatic EIS was performed with a BioLogic MTZ-35 impedance analyzer (3.7 MHz to 1 Hz with a 10 mV perturbation). The impedance spectra were fitted using an equivalent circuit model in the ZView software package. For the critical current density test, Li/LLZTO/Li cells were cycled with a BioLogic VMP3 potentiostat at 30 °C and 3 MPa stack pressure.

## Supporting Information Available

XPS survey spectrum of LLZTO, additional O 1s and C 1s XPS data, additional EIS data, and LLZTO XRD spectrum (PDF)
