# Supplementary material for: Removal and Reoccurrence of LLZTO Surface Contaminants under Glovebox Conditions
Source: ACS Appl Mater Interfaces. 2024 May 16;16(21):27230–41. doi: 10.1021/acsami.4c00444 (PMC11145597; doi:10.1021/acsami.4c00444)
Supplement: Supplementary file 1 — am4c00444_si_001.pdf [file am4c00444_si_001.pdf]

Supporting Information

Removal and Reoccurrence of LLZTO Surface Contaminants Under Glovebox Conditions

Marco Siniscalchi,[*,†,‡] Joshua S. Gibson,[†,¶] James Tufnail,[†] Jack E. N. Swallow,[†] Jarrod Lewis,[†] Guillaume Matthews,[†] Burcu Karagoz,[§] Matthijs A. van Spronsen,[§] Georg Held,[§] Robert S. Weatherup,[†,‡] Chris R. M. Grovenor,[†,‡] and Susannah C. Speller[*,†]

†*Department of Materials, University of Oxford, Oxford, OX1 3PH, UK*

‡*The Faraday Institution, Didcot, OX11 0RA, UK*

¶*School of Chemistry, University of Edinburgh, Edinburgh, EH9 3FJ*

§*Diamond Light Source, Didcot, OX11 0DE, UK*

E-mail: marco.siniscalchi@materials.ox.ac.uk; susannah.speller@materials.ox.ac.uk

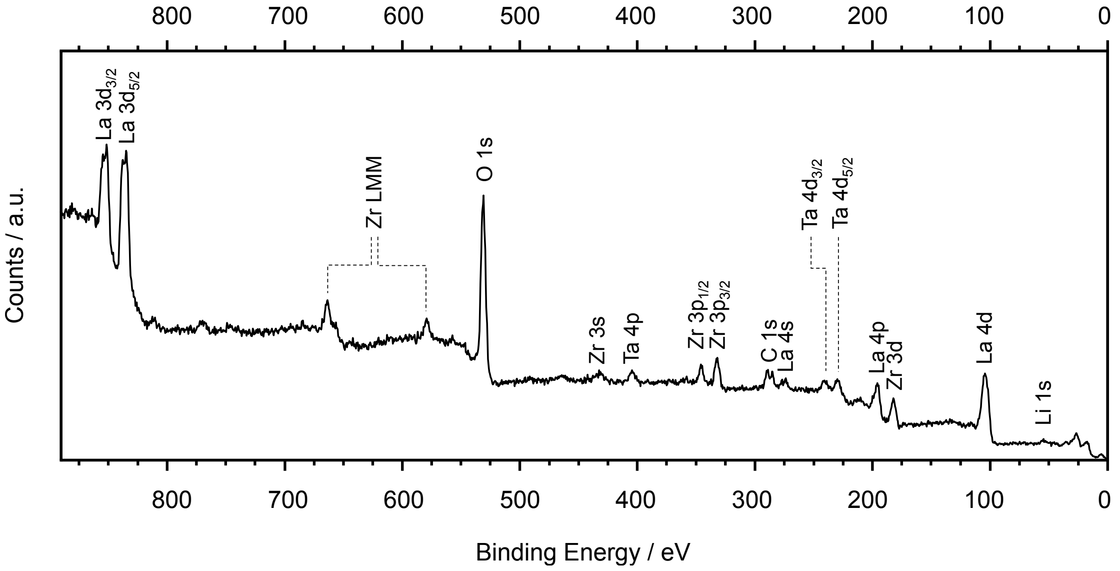

Figure S1: XPS survey spectrum of an LLZTO pellet after polishing in air. No unfamiliar contamination is seen on the pellet surface. The spectrum was collected with a 2500 eV incident photon energy.

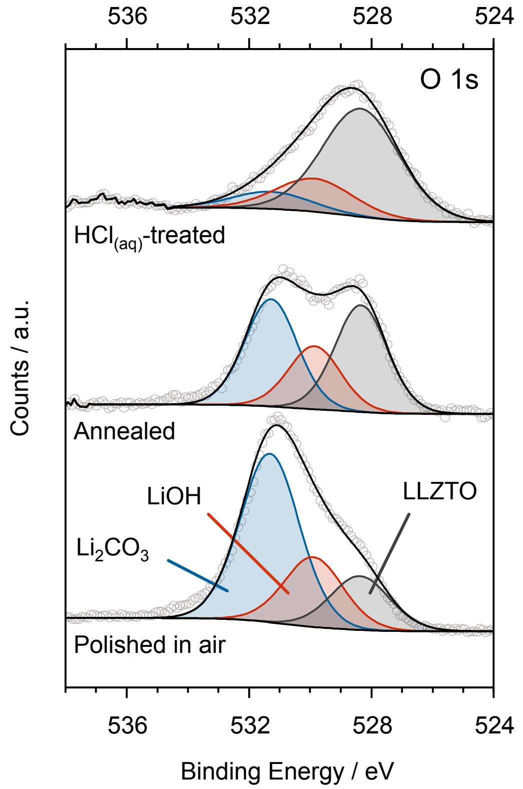

Figure S2: XPS O 1s spectra of LLZTO pellets after different surface treatments. The acid treatment in $HCl_{(aq)}$ is superior in removing the surface contaminants Li_2CO_3 and LiOH. The broader peaks for the acid-treated sample likely originate from a reduced electronic charge compensation.

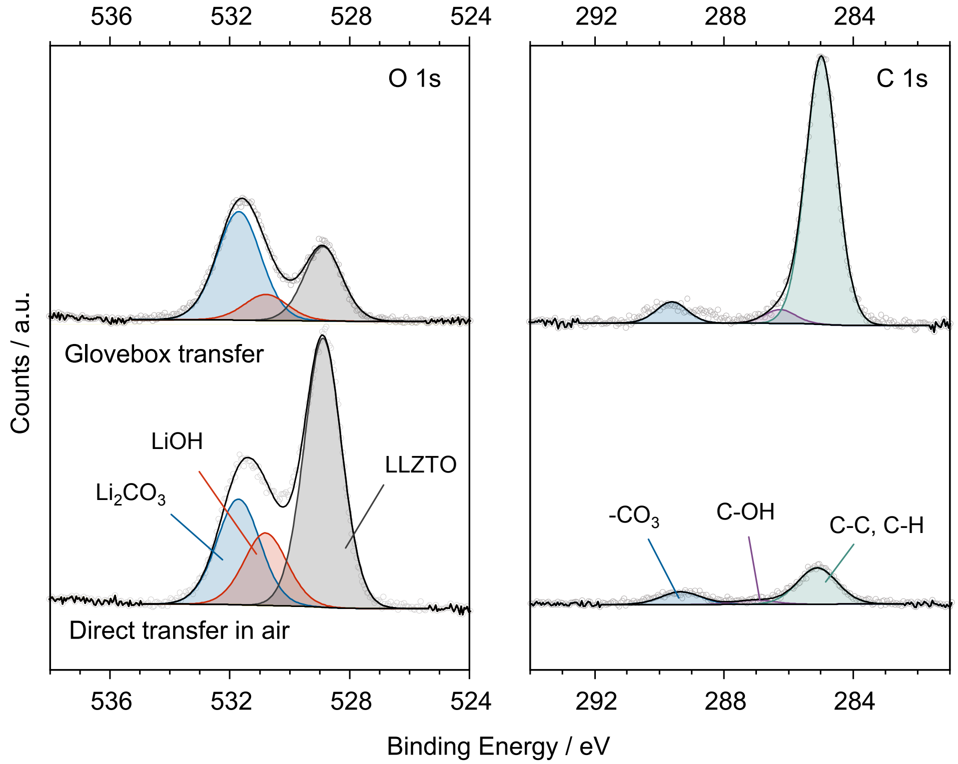

Figure S3: XPS O 1s and C 1s spectra collected from LLZTO pellets after acid treatment in $HCl_{(aq)}$. For the bottom spectra, the LLZTO was immediately loaded in the XPS ($<$ 1 min) after the surface treatment in air. For the top spectra, the LLZTO pellet was promptly loaded in the glovebox ($<$ 1 min) after the surface treatment, and then transferred to the XPS in a sealed vessel (ULVAC Phi GmbH). The LLZTO that was loaded via the glovebox exhibits significantly greater adventitious carbon signal and reacted oxide surface contamination. For this experiment, we used a lab-based ULVAC Phi Versaprobe III XPS with a monochromatic Al K_α source (hν 1486.6 eV, 15 kV anode voltage, 25 W beam power).

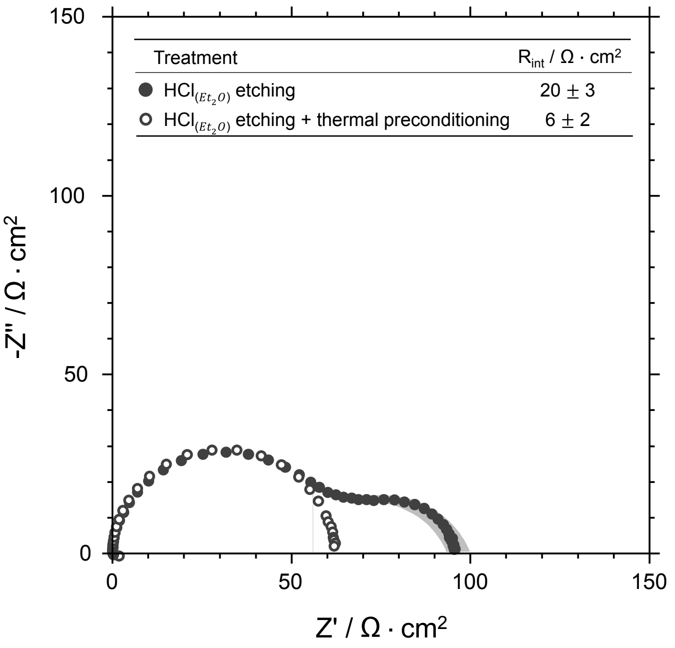

Figure S4: After acid etching of the LLZTO pellet, a thermal preconditioning of the Li/LLZTO/Li cell at ~ 170 °C under approximately 2 MPa of pressure for 2 hours results in a further decrease in the Li/LLZTO interfacial impedance.

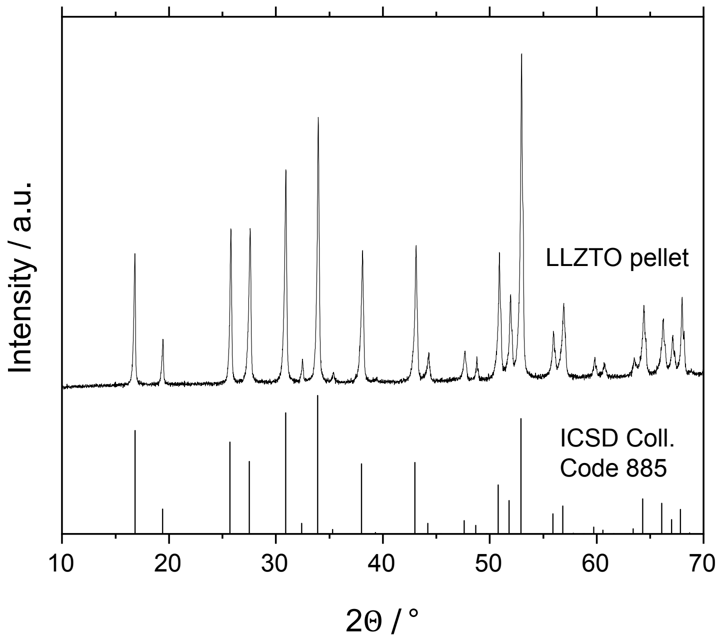

Figure S5: X-ray diffraction of the LLZTO pellets used in this study. The expected peaks are observed and no contamination could be detected.
